# Cost-effectiveness of Web-Based and Home-Based Postnatal Psychoeducational Interventions for First-time Mothers: Economic Evaluation Alongside Randomized Controlled Trial

**DOI:** 10.2196/25821

**Published:** 2022-03-11

**Authors:** Qishi Zheng, Luming Shi, Lixia Zhu, Nana Jiao, Yap Seng Chong, Sally Wai-Chi Chan, Yiong Huak Chan, Nan Luo, Wenru Wang, Honggu He

**Affiliations:** 1 Epidemiology Singapore Clinical Research Institute Singapore Singapore; 2 Cochrane Singapore Singapore Singapore; 3 Duke-NUS Medical School Singapore Singapore; 4 Alice Lee Centre for Nursing Studies Yong Loo Lin School of Medicine National University of Singapore Singapore Singapore; 5 National University Health System Singapore Singapore; 6 Edson College of Nursing and Health Innovation Arizona State University Arizona, AZ United States; 7 Division of Maternal Fetal Medicine Department of Obstetrics & Gynaecology National University Hospital Singapore Singapore; 8 Yong Loo Lin School of Medicine National University of Singapore Singapore Singapore; 9 Tung Wah College Hong Kong China; 10 Biostatistics Unit National University of Singapore Singapore Singapore; 11 Saw Swee Hock School of Public Health National University of Singapore Singapore Singapore

**Keywords:** anxiety, cost-effectiveness, depression, first-time mother, home-based, postnatal, psychoeducational, self-efficacy, social support, web-based

## Abstract

**Background:**

The cost-effectiveness of interventions has attracted increasing interest among researchers. Although web-based and home-based psychoeducational interventions have been developed to improve first-time mothers’ postnatal health outcomes, very limited studies have reported their cost-effectiveness.

**Objective:**

The aim of this study was to evaluate the cost-effectiveness of web-based and home-based postnatal psychoeducational interventions for first-time mothers during the early postpartum period.

**Methods:**

A randomized controlled 3-group pretest and posttest design was adopted, and cost-effectiveness analysis from the health care’s perspective was conducted. A total of 204 primiparas were recruited from a public tertiary hospital in Singapore from October 2016 to August 2017 who were randomly allocated to the web-based intervention (n=68), home-based intervention (n=68), or control (n=68) groups. Outcomes of maternal parental self-efficacy, social support, postnatal depression, anxiety, and health care resource utilization were measured using valid and reliable instruments at baseline and at 1 month, 3 months, and 6 months after childbirth. The generalized linear regression models on effectiveness and cost were used to assess the incremental cost-effectiveness ratios of the web-based and home-based intervention programs compared to routine care. Projections of cumulative cost over 5 years incurred by the 3 programs at various coverage levels (ie, 10%, 50%, and 100%) were also estimated.

**Results:**

The web-based intervention program dominated the other 2 programs (home-based program and routine care) with the least cost (adjusted costs of SGD 376.50, SGD 457.60, and SGD 417.90 for web-based, home-based, and control group, respectively; SGD 1=USD 0.75) and the best improvements in self-efficacy, social support, and psychological well-being. When considering the implementation of study programs over the next 5 years by multiplying the average cost per first-time mother by the estimated average number of first-time mothers in Singapore during the 5-year projection period, the web-based program was the least costly program at all 3 coverage levels. Based on the 100% coverage, the reduced total cost reached nearly SGD 7.1 million and SGD 11.3 million when compared to control and home-based programs at the end of the fifth year, respectively.

**Conclusions:**

The web-based approach was promisingly cost-effective to deliver the postnatal psychoeducational intervention to first-time mothers and could be adopted by hospitals as postnatal care support.

**Trial Registration:**

ISRCTN registry ISRCTN45202278; https://www.isrctn.com/ISRCTN45202278

## Introduction

The early postpartum period is a stressful transition period for first-time mothers owing to physical and emotional challenges related to childbirth and challenges in adapting to their new social roles, for example, parenting newborns and establishing mother-infant relationship [1,2]. Based on the maternal role attainment theory, a mother’s emotional health during the postpartum period is mainly influenced by the mother’s biopsychosocial well-being, family, and her surrounding environment [3]. Many first-time mothers feel inadequately prepared for motherhood and are situated in environments with insufficient support [4-6].

Psychoeducational interventions have been developed to improve new mothers’ knowledge in self-care and newborn care during the postnatal period and to build on their strengths and resources to promote emotional coping and parenting skill development [5,7]. In Asian countries, including Singapore, the follow-up home visits undertaken by midwives are not commonly practiced [8]. Home-based psychoeducation intervention via home visits is not easily accessible owing to the shortage of midwives and nurses, and its cost-effectiveness was undetermined [5,6,8]. Conversely, a similar intervention delivered online, for example, a web-based platform, could address the issues of inaccessibility and high cost [9,10]. About two-thirds of the world population (62%) had access to the internet in 2020 compared to 5.9% in 2000 [11]. In Singapore, a newly initiated Home Access Program allows lower-income households to afford fiber broadband connectivity and a tablet at a subsidized rate to increase the population’s internet accessibility [12,13], thus allowing more people to access web-based resources. Moreover, the accessibility to web-based interventions may significantly reduce people’s reliance on health care professionals, thus lowering the burden on health care, that is, administrative cost, time, and resource savings [11]. Therefore, if the web-based intervention is effective, it has the potential to be highly cost-effective as it can be delivered at scale across large populations, with relatively low additional costs per additional user [14] unlike face-to-face education or in-person visits where labor costs account for a substantial proportion of the total cost [15].

Although the effectiveness of web-based and mobile app–based interventions [16-18] have been reported, limited information exists on their cost-effectiveness. Previous studies have revealed that compared to routine care or face-to-face intervention, web-based or internet-based intervention could be a cost-effective way for alcohol prevention in adolescents [11], increasing physical activity for healthy adult Latina women [9], dietetic services for weight management [19], and self-management for people with type 2 diabetes [20]. However, there is no study reporting the cost-effectiveness of web-based and home-based psychoeducational interventions for first-time mothers. Such knowledge may help to keep costs to a minimum and make the intervention affordable for all mothers regardless of their socioeconomic status, while achieving positive maternal outcomes.

We conducted a 3-group randomized controlled trial that aimed to examine the effectiveness and cost-effectiveness of web-based and home-based postnatal psychoeducational programs in first-time mothers. The details of the interventions have been reported in the study protocol [12], and the outcomes of their effectiveness have been reported in a separate paper [16]. This paper aims to evaluate the cost-effectiveness of web-based and home-based postnatal psychoeducational interventions on outcomes of self-efficacy in newborn care (primary outcome), social support, postnatal depression, and anxiety (secondary outcomes) in first-time mothers during the early postpartum period.

## Methods

### Ethics Approval

This study was approved by the National Health Group Domain Specific Review Board on 25 January 2016 (NHG DSRB Ref: 2015/01189).

### Study Design, Setting, and Sample

A randomized controlled trial was carried out in a tertiary public hospital in Singapore. First-time mothers recruited from the postnatal wards were randomly allocated to one of the 3 intervention groups: web-based group (receiving web-based psychoeducational intervention plus routine care), home-based group (receiving home-based psychoeducational intervention plus routine care), or control group (receiving routine care). Mothers were excluded if they had identified physical or mental disorders before and during pregnancy, had complicated assisted delivery with fourth-degree perineal tear, gave birth to a stillborn child, or had a child with a congenital anomaly or medical complications [8]. The sample size of 204 was calculated based on the primary outcome of maternal parental self-efficacy, with 68 allocated in each group [12,16]. The recruitment period was from October 2016 to February 2017, and the last 6 months follow-up data were collected in August 2017. The study design and procedure have been previously reported in papers detailing the study protocol [12] and clinical effectiveness [16]. This study conducted a cost-effectiveness analysis from the health care provider’s perspectives alongside the randomized controlled trial.

### Interventions

#### Control Group

Participants allocated to the control group only received routine care provided by the hospital. It involved postnatal support from nurses and midwives in the hospital and a postdelivery follow-up (around 1-6 weeks) outpatient appointment with the doctor. The support focused on providing didactic information on basic baby care tasks while mothers were hospitalized for childbirth as well as the inspection of episiotomy or cesarean section wound and breastfeeding advice during the follow-up hospital visit.

#### Home-Based Intervention Group

Participants allocated to the home-based group received a 1-hour face-to-face postnatal psychoeducational intervention via a home visit by a registered nurse in addition to routine care. A self-developed educational booklet [12,16] developed based on the evidence of literature by Shorey et al (unpublished data, 2012) and Wong et al [21] was used to facilitate the face-to-face intervention. The booklet was provided to the participants before hospital discharge. Three weekly telephone calls were included in the intervention to address mothers’ queries. The postnatal psychoeducational intervention contents were developed based on Bandura’s self-efficacy theory [22], social support model, findings from preliminary studies [4,5], and previous literature [23-26].

#### Web-Based Intervention Group

**Figure 1:**
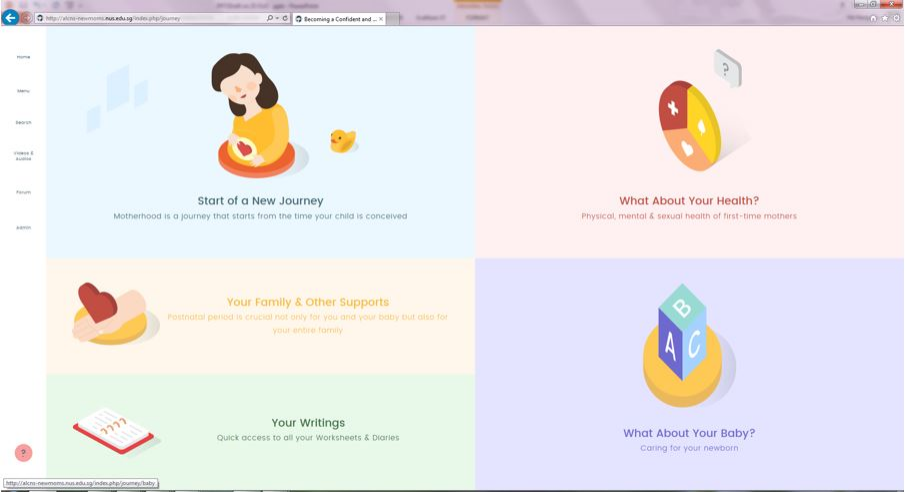
Homepage of the website for mothers in the web-based intervention group.

**Figure 2:**
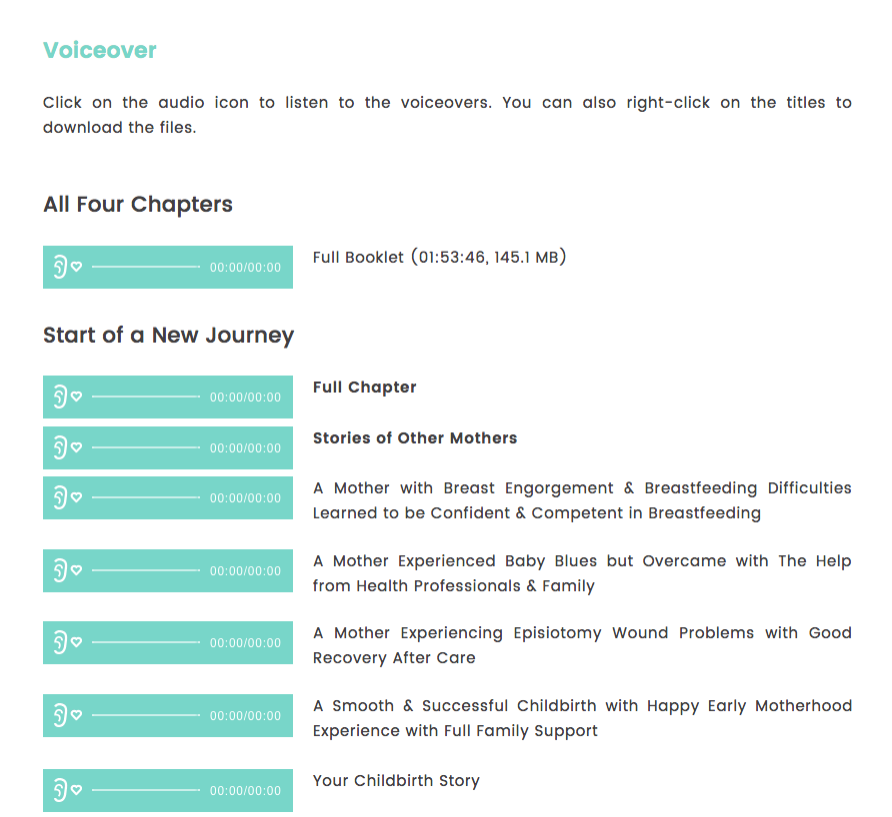
Sample page of voiceover of the contents on the website.

Briefly, participants in the web-based group received a postnatal psychoeducational intervention via a newly developed website (Figure 1) with 1-month online access after childbirth in addition to routine care. The main contents on the website were identical to those in the booklet, with section-to-section audio files (Figure 2) provided for the mothers’ convenience. Videos demonstrating breastfeeding techniques, breast engorgement management, infant bathing, and Kegel exercises based on current practices in local hospitals were also developed and embedded on the website. A peer discussion forum and a confidential corner for the participants to communicate with other mothers or research team members were also available. Three weekly telephone calls, each lasting about 3 minutes, served as reminders for participants to access the website; participants did not receive additional information.

### Health Outcomes and Instruments

The health outcomes used to measure the effectiveness of the interventions included maternal parental self-efficacy, social support, postnatal depression, and anxiety. The instruments used included the 17-item Perceived Maternal Parental Self-efficacy Scale (total score range 17-68, higher scores meaning better self-efficacy) [27,28], the 22-item modified Perinatal Infant Care Social Support Scale (total score range 22-88, higher scores indicating better social support) [28,29], the 10-item Edinburgh Postnatal Depression Scale (total score range 0-30, lower scores meaning less depressive symptoms) [30], and the Anxiety subscale of the Hospital Anxiety and Depression Scale (total score range 0-21, lower scores meaning less anxious) [31]. The higher scores of self-efficacy and social support and the lower scores of depression and anxiety indicated better outcomes. Detailed descriptions of the instruments and their validity and reliability can be found in papers previously published for this project [12,16].

### Costs and Health Care Resource Utilization

The costs referred to in this study were viewed from a health care provider’s perspective, which included costs associated with the study interventions and costs related to the health service utilization to treat postnatal medical conditions in mothers and babies. Broadly, costs associated with the study interventions included those incurred during the development and implementation of interventions, ongoing delivery, and maintenance of the interventions. Costs related to the development and implementation of the web-based intervention include the purchasing of goods and services for hardware, software, educational materials, webpage design, maintenance, and work delivered by third-party service providers and were recorded from actual invoices. Costs of delivering the home-based intervention program were captured based on home visit human resources, material costs, and transportation fees.

Costs associated with health service utilization due to postnatal medical conditions were collected using the Questionnaire on Healthcare Service Utilization during 3 periods: within 1 month after childbirth, 1-3 months after childbirth, and 3-6 months after childbirth. All medical conditions that required health care services were captured and classified into 2 categories: infant-related conditions and maternal-related conditions. Service provider information was also recorded, which included government polyclinics, private clinics (including general practitioner clinics), hospital specialist outpatient clinics, hospital emergency departments, hospital inpatient admissions, over-the-counter pharmacies, and others. For every health service received by patients, data on full bills (ie, before any subsidies or insurance payments) were collected to estimate the health care provider’s full cost for each service. In Singapore, the public health care delivery system is not-for-profit. The entire bill size is the best estimate for the true cost incurred for delivering care. The entire bill size for certain health care services is not charged at a flat rate; it varies among patients depending on the duration of care delivery at each visit and the seniority of the personnel attending to the patient. For example, the cost of an outpatient visit is higher if the consultation time with a senior consultant is longer compared to a shorter consultation time with a junior consultant, although the unit cost for the medications and procedures is standardized.

### Data Analysis

#### Costs Associated With Study Interventions

Costs related to development and implementation of the web-based intervention were further converted to an annualized average cost per mother by assuming that the website could be used for 5 years [32]. We also considered a depreciation factor at 3% for the calculation of the above costs. Costs related to delivering home-based interventions were averaged for each participating patient in the study group as well. Since all 3 groups received identical routine care, the costs associated with standard care were not included in the cost-effectiveness analysis.

#### Costs Associated With Postnatal Health Services

For postnatal health services related to mothers’ and infants’ medical conditions, the costs were summarized into 3 categories: service providers, infant-related conditions, and maternal-related conditions. For each health service, for example, the general practitioner and private clinic, the average cost per visit was estimated by dividing the total bills incurred from patients who utilized this service during the study period by the total number of such visits. All costs were calculated in SGD (2016-2017, SGD 1=USD 0.75).

#### Base Case Analysis

The base case analysis included all participants with complete follow-up information and original randomized allocation (N=193). The generalized linear regression models on effectiveness and cost at the sixth month were applied to assess the cost-effectiveness of the 3 programs, adjusting for relevant sociodemographic and clinical characteristics. Univariable models were first fit to identify the potential confounding factors associated with effectiveness and cost. For example, the effectiveness model was adjusted for potential confounding factors such as baseline values, age, number of days since baseline, ethnicity, employment status, monthly household income, prenatal courses attendance, and skin-to-skin contact with baby. The cost model was adjusted for factors that might influence the utilization of health and other services (ie, employment status and income levels). These adjustments aimed to obtain average effectiveness and cost accounting for the influence from abovementioned risk factors instead of the crude results that might not be representative. To account for mothers with zero cost, that is, those who did not consume any health care resources during the study period, a 2-part model approach was adopted. The first-part probit model estimated the probability of zero cost and the second-part generalized linear regression model, with a log link in gamma family, handled nonzero positive cost data. For the base case analysis of cost-effectiveness, time horizon for the health outcomes and costs associated with ongoing delivery and maintenance of the interventions were consistent with the randomized controlled trial, that is, 6 months, with no discounting applied. Incremental cost-effectiveness ratios (ICERs) were estimated to measure the economic value of the study interventions in comparison to the control group. It was calculated by dividing the difference in total costs (incremental cost) by the difference in health outcomes or effects at 6-month postintervention (incremental effect) to provide a ratio of extra cost per unit of incremental health effect. When calculating the ICER, the depression and anxiety scores were inverted, which means that the higher the score, the better after inversion.

#### Sensitivity Analyses

Probabilistic sensitivity analyses were adopted to address the parameter uncertainty, that is, cost and effectiveness. The distribution of the resulting 10,000 estimates of the ICER on the cost-effectiveness plane depicts a joint uncertainty surrounding costs and outcomes. Estimates in the lower right quadrant of a cost-effectiveness plane suggest that the program is more effective and less costly, while estimates in the upper right quadrant suggest that it is more effective but costlier. The cost-effectiveness acceptability curve was used to display the probability that 1 program is cost-effective at a given willingness-to-pay threshold. Several scenario analyses were conducted to re-estimate the ICER to explore the results’ sensitivity to particular assumptions or parameters: (1) to assume web-based and home-based program would be used for only 3 years and (2) to vary the overall program cost from 0% to 300%.

### Projection of Nationwide Implementation of Interventions Over 5 Years

The economic impact of national implementation of study interventions over the next 5 years was estimated by multiplying the average cost per the first-time mother by the estimated average number of first-time mothers in Singapore. The number of first-order babies born from 2012 to 2016 in Singapore ranged from 19,292 to 20,755, yielding an average of 20,000 first-order babies each year [33]. In our projection, we assumed that all 20,000 first-time mothers would be eligible for the psychoeducational program. To consider different acceptance levels of interventions, 3 program coverage levels, that is, 10%, 50%, and 100% for the eligible first-time mothers were adopted for economic impact analysis.

## Results

### Characteristics of the Participants and the Baseline Health Outcomes Among the 3 Groups

In total, 204 mothers were recruited, with 193 included in the economic evaluation (64 for web-based, 63 for home-based, and 66 for control group). The CONSORT diagram and details of the mothers’ characteristics have previously been published [16]. Briefly, the mean age of the mothers was around 30 years for all 3 groups, and half of them were of Chinese ethnicity. Over 80% of the first-time mothers obtained a bachelor’s degree or above (165/204) and were employed (175/204, 85%) during the study period. Nearly three-fourths of the mothers had monthly household incomes of more than SGD 5000 and the majority (178/204, 87%) were healthy and had no chronic diseases. Overall, around 58% (119/204) of the first-time mothers had normal deliveries. There were no statistically significant differences in all sociodemographic, clinical, and baseline data among mothers in the 3 groups. There were no significant differences in the baseline outcomes of self-efficacy, social support, postnatal depression, and anxiety among the 3 groups [16].

### Costs

During the 6-month follow-up period, health services utilization data seeking various services concerning infant-related and maternal-related conditions showed similar occurrence rates among the 3 groups (Table 1). The number and percentage of zero–health service use mothers were as follows: web-based (28/64 44%), home-based (20/63, 32%), and control (30/66, 46%). The average health care cost per participant was SGD 339.90 (SD 493) for the web-based group, SGD 375.40 (SD 699.9) for the home-based group, and SGD 411.20 (SD 601) for the control group. Moreover, the web-based program involved an upfront cost of SGD 17,210 to develop both the web design and educational videos and an annual web maintenance fee of SGD 2900 per year. Similarly, the home-based program required a SGD 233.40 upfront one-time training fee for the payment of an experienced Registered Midwife to train the Registered Nurse who delivered the face-to-face postnatal psychoeducation; home visits cost SGD 77.42 for 1-hour session, which is inclusive of the manpower cost as well as the costs of printed materials and transportation. When accounting for all relevant costs, including 6 months’ health care utilization cost, the web-based program cost an average of SGD 390.80/participant, while home-based and control programs cost SGD 437.80/participant and SGD 411.20/participant, respectively.

**Table 1:** Service use during the follow-up period (N=193).

		Web-based intervention (n=64)^a^			Home-based intervention (n=63)^b^			Control (routine care) (n=66)^c^	
		n	Cost (SGD)^d^, mean (SD)	n		Cost (SGD), mean (SD)	n		Cost (SGD), mean (SD)
**Service providers**									
	General practitioner and private clinic	43	127 (99.28)	44		150 (171.72)	41		260 (400.52)
	Hospital outpatient	17	205 (201.90)	21		264 (241.48)	34		208 (193.93)
	Hospital accident and emergency	25	199 (197.84)	24		392 (990.39)	16		276 (600.57)
	Hospital inpatient	6	771 (452.60)	2		200 (0)	8		968 (744.45)
	Polyclinic	2	149 (148.49)	0		0	4		38 (25.63)
	Over-the-counter	0	0 (0)	2		18 (2.83)	1		20 (0)
	Others	19	179 (185.07)	13		92 (67.15)	6		155 (163.28)
**Infant-related conditions**									
	Spit up (milk)	12	196 (125.84)	6		198 (174.98)	13		110 (108.66)
	Excessive eye discharge	5	156 (61.81)	1		55 (0)	2		519 (536.23)
	Infantile eczema	3	76 (70.85)	5		96 (78.46)	12		217 (270.02)
	Diaper rash	4	74 (36.77)	1		34 (0)	4		180 (195.25)
	Others	48	239 (287.20)	54		244 (613.23)	43		298 (492.38)
	Total	72	215 (248.68)	67		248 (617.18)	74		255 (409.74)
**Maternal-related conditions**									
	Insufficient milk supply	13	128 (192.12)	8		100 (83.47)	5		91 (30.61)
	Breast engorgement	9	139 (114.95)	7		145 (98.33)	7		175 (144.01)
	Mastitis	3	94 (70.06)	10		282 (351.09)	2		56 (61.77)
	Prolonged lochia	1	30 (0)	0		0 (0)	1		20 (0)
	Others	14	219 (231.55)	15		179 (134.66)	21		351 (496.33)
	Total	40	157 (186.14)	40		183 (204.62)	36		257 (403.54)

^a^Upfront cost: SGD 17,210, follow-up cost: SGD 2900 per year, average total cost per participant (a depreciation factor 3% over 5 years was considered for the average total cost per participant calculation): SGD 390.8, average health care cost: SGD 339.9 (SD 493).

^b^Upfront cost: SGD 233.4, follow-up cost: SGD 77.42 per visit, average total cost per participant (a depreciation factor 3% over 5 years was considered for the average total cost per participant calculation): SGD 437.8, average health care cost: SGD 375.4 (SD 699.9).

^c^Upfront cost: SGD 0, follow-up cost: SGD 0, average total cost per participant (a depreciation factor 3% over 5 years was considered for the average total cost per participant calculation): SGD 411.2, average health care cost: SGD 411.2 (SD 601).

^d^SGD 1=USD 0.75.

### Cost-effectiveness Analyses Results

The effectiveness of the web-based and home-based psychoeducational intervention programs has previously been reported [16]. Point estimates of the ICER for the scores of self-efficacy, social support, postnatal depression, and anxiety are reported in Table 2. In Table 2, the adjusted effectiveness of each outcome (self-efficacy, social support, depression, and anxiety) was estimated by adjusting for baseline values, age, number of days since the baseline, ethnicity, employment status, monthly household income, prenatal courses attendance, and skin-to-skin contact with the baby. The base-case analysis showed that the web-based group incurred the least cost and was the most effective among the 3 groups across all outcome measures. It saved around SGD 81 per participant (18% of the average adjusted cost) compared to the home-based program (adjusted cost: web-based group SGD 376.50 vs home-based group SGD 457.60) while showing better performance in all the outcomes, and SGD 41 per participant compared to the control group (adjusted cost: web-based group SGD 376.50 vs control group SGD 417.90) with better performance in all the outcomes. Hence, it was considered dominant across all the outcomes. When comparing the home-based group to the control group, the ICER ranged from SGD 8.60 per social support score improvement to SGD 90.20 per anxiety score (Anxiety subscale of the Hospital Anxiety and Depression Scale) reduction. When the validity of the programs was set for 3 years, the web-based program remained the dominant strategy to provide postnatal care (Table 2). The same pattern was observed when the overall program cost was varied at 0% and 50%. At 200% and 300% of the original overall program costs, the control program became the most cost-effective strategy, followed by the web-based and home-based programs.

**Figure 3:**
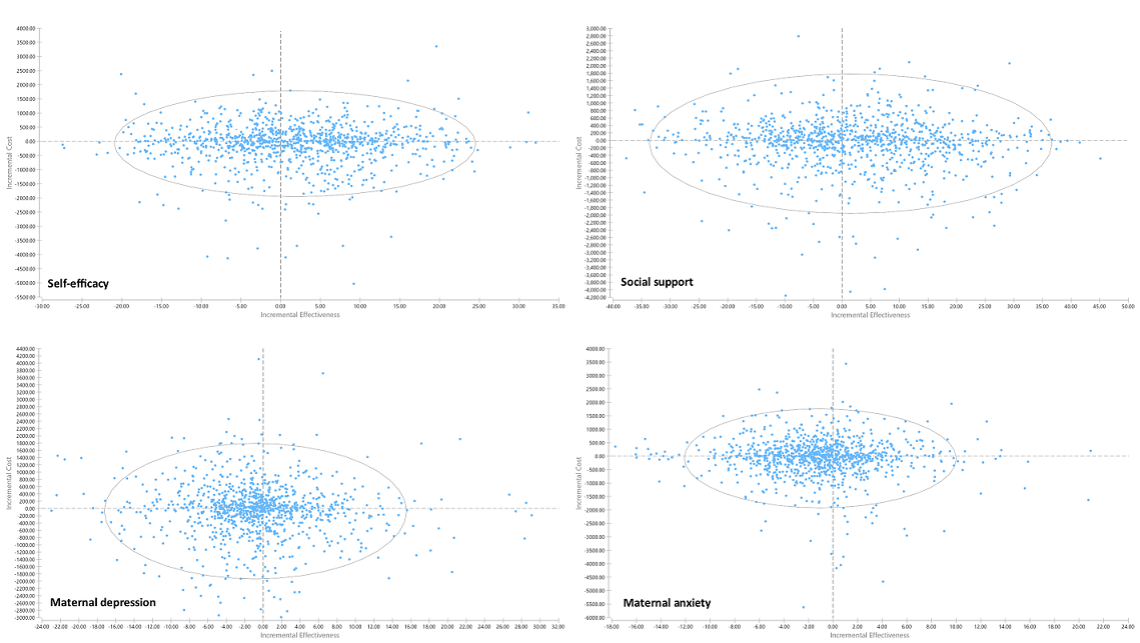
Cost-effectiveness planes of web-based versus home-based groups (home-based as the base for the incremental calculation) for all outcomes (favored the positive side for all outcomes, the scores of depression and anxiety had been inverted when calculating the incremental cost-effectiveness ratio) (N=193).

**Figure 4:**
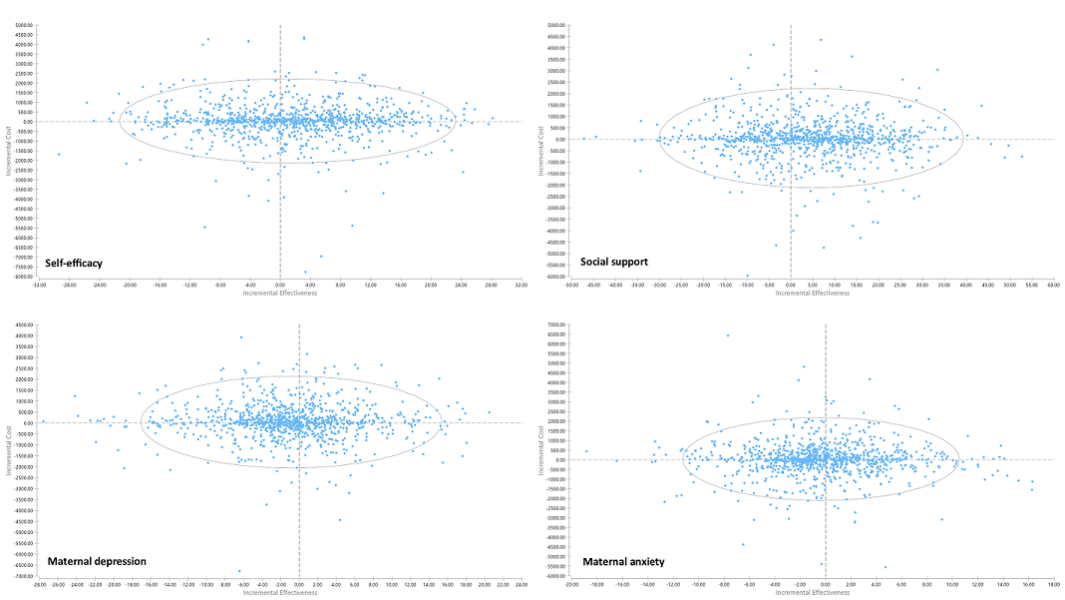
Cost-effectiveness planes of home-based versus control groups (control as the base for the incremental calculation) for all outcomes (favored the positive side for all outcomes, the scores of depression and anxiety had been inverted when calculating the incremental cost-effectiveness ratio) (N=193).

**Figure 5:**
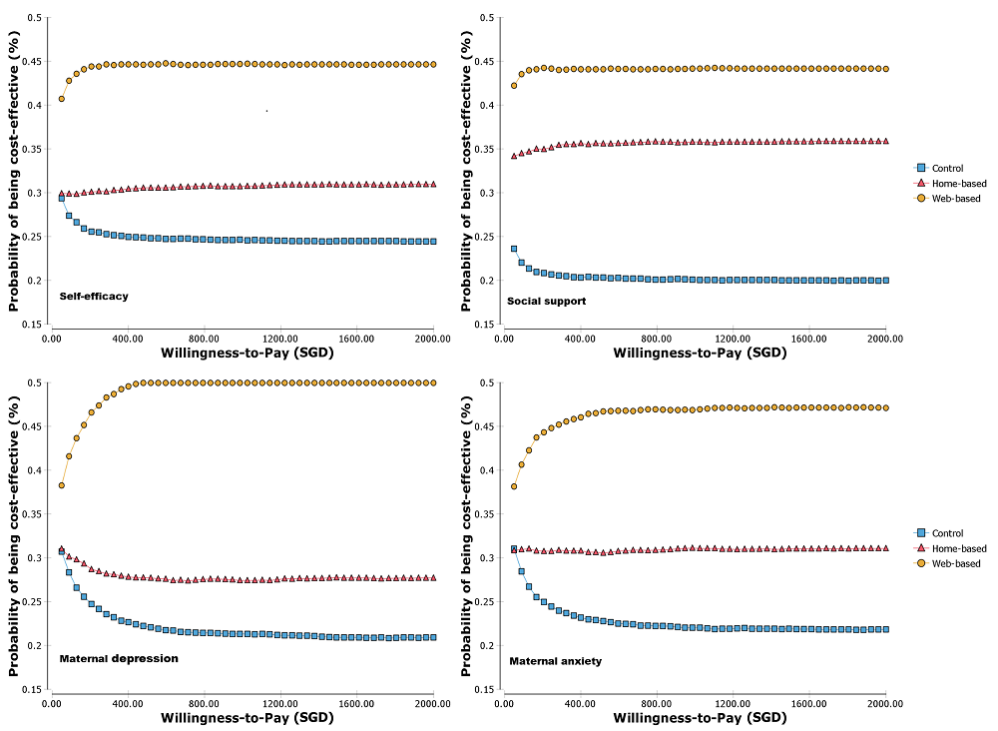
Cost-effectiveness acceptability curves for all outcomes at various willingness-to-pay thresholds (per score improvement) among the 3 groups (N=193).

A probabilistic sensitivity analysis was adopted to account for uncertainty from the trial. Cost-effectiveness planes comparing web-based versus home-based and home-based versus control were also generated (Figures 3 and 4). As shown in Figure 3, the ICER estimates for self-efficacy and social support were more in the right-side quadrants when comparing web-based versus home-based interventions, and the ICER estimates for maternal depression and anxiety were more in the left-side quadrants. Similar patterns were observed for the comparison of home-based versus control groups (Figure 4). The cost-effectiveness acceptability curves (Figure 5) showed the probability of cost-effectiveness for the 3 groups at various willingness-to-pay levels for the scores of self-efficacy, social support, postnatal depression, and anxiety. The pattern of the cost-effectiveness acceptability curves for the self-efficacy, social support, postnatal depression, and anxiety scores were similar, with the highest curve from the web-based program and the lowest curve from the control program. As the web-based program was the most effective and was associated with the lowest average total cost among the 3 programs, it was most likely to be cost-effective at all willingness-to-pay levels. However, its probability of cost-effectiveness showed a flat trend when the willingness-to-pay level reached around SGD 100 per self-efficacy or per social support (per score change on the scale) and SGD 400 per maternal depression or per maternal anxiety (per score change on the scale). The lowest curve for all 4 outcomes was for the control group, which indicated that the routine care was the least likely to be cost-effective than the other 2 intervention programs.

**Table 2:** Incremental cost-effectiveness ratios from the base case and different scenarios for various outcome measures (N=193).

Scenario, group			Adjusted cost (SGD)^a^		Self-efficacy (PMPSE)^b^				Social support (PICSS-modified)^c^					Depression (EPDS)^d^					Anxiety (HADS-A)^e^		
					AE^f^	ICER^g^		AE			ICER		AE			ICER^h^		AE			ICER^h^
**Base case**																					
	W^i,j^	376.50		59.11		Dominant^k^	81.6			Dominant		4.44			Dominant		*3.36*			Dominant	
	C^l^	417.90		56.28		N/A^m^	75.3			N/A		6.17			N/A		4.81			N/A	
	H^n^	457.60		57.27		40.1	79.9			8.6		5.45			55.1		4.37			90.2	
**Program valid for 3 years only**																					
	W^i^	392.90		59.11		Dominant	81.6			Dominant		4.44			Dominant		*3.36*			Dominant	
	C	417.90		56.28		N/A	75.3			N/A		6.17			N/A		4.81			N/A	
	H	458.20		57.27		40.7	79.9			8.8		5.45			56.0		4.37			91.6	
**0% of the overall program cost**																					
	W^i^	325.55		59.11		Dominant	81.6			Dominant		4.44			Dominant		*3.36*			Dominant	
	H	380.22		56.28		Dominated^o^	75.3			Dominated		6.17			Dominated		4.81			Dominated	
	C	417.90		57.27		Dominated	79.9			Dominated		5.45			Dominated		4.37			Dominated	
**50% of the overall program cost**																					
	W^i^	351.01		59.11		Dominant	81.6			Dominant		4.44			Dominant		*3.36*			Dominant	
	C	417.90		56.28		Dominated	75.3			Dominated		6.17			Dominated		4.81			Dominated	
	H	419.12		57.27		Dominated	79.9			Dominated		5.45			Dominated		4.37			Dominated	
**200% of the overall program cost**																					
	C^i^	417.90		59.11		N/A	81.6			N/A		4.44			N/A		*3.36*			N/A	
	W	427.38		56.28		3.3	75.3			*1.5*		6.17			5.5		4.81			6.5	
	H	535.81		57.27		Dominated	79.9			Dominated		5.45			Dominated		4.37			Dominated	
**300% of the overall program cost**																					
	C	417.90		59.11		N/A	81.6			N/A		4.44			N/A		*3.36*			N/A	
	W	478.29		56.28		21.3	75.3			9.6		6.17			34.9		4.81			*41.6*	
	H	613.61		57.27		Dominated	79.9			Dominated		5.45			Dominated		4.37			Dominated	

^a^SGD 1=USD 0.75.

^b^PMPSE: Perceived Maternal Parental Self-Efficacy.

^c^PICSS-modified: modified Perinatal Infant Care Social Support.

^d^EPDS: Edinburgh Postnatal Depression Scale.

^e^HADS-A: Anxiety subscale of the Hospital Anxiety and Depression Scale.

^f^AE: adjusted effectiveness.

^g^ICER: incremental cost-effectiveness ratio.

^h^The scores of depression/anxiety were inverted before calculating the incremental cost-effectiveness ratio so that the higher scores indicate the better after inversion.

^i^The group with the least cost in each scenario is italicized.

^j^W: web-based intervention group.

^k^Dominant means the program is the best in both cost and efficacy among the 3 groups.

^l^C: control group.

^m^N/A: not applicable.

^n^H: home-based intervention group.

^o^Dominated means the program is the worst in both cost and efficacy among the 3 groups.

### Projection of Nationwide Implementation of Interventions Over 5 Years

As shown in Table 3, during the 5-year projection period, the web-based intervention was the least costly program at all 3 coverage levels (ie, SGD 3,430,711 at 10% coverage, SGD 17,026,711 at 50%, and SGD 34,021,711 at 100%), and based on the 100% coverage, the reduced total cost reached nearly SGD 7.1 million (41,120,000 minus 34,021,711) and SGD 11.3 million (45,282,233 minus 34,021,711) compared to the control and home-based groups at the end of the fifth year, respectively. Although there was a relatively high upfront cost for the web-based program, it needed a very low maintenance fee for each year after the first year and provided almost unlimited concurrent access to the web-based contents for all target populations. On the contrary, the home-based program required intensive manpower to deliver face-to-face teaching for each visit, which was sensitive to the program’s coverage and comprised 17% of the total cost. Therefore, even with the lower health care cost over 5 years, the home-based program was the costliest in terms of the total cost incurred based on the base-case analysis results.

**Table 3:** Projections of cumulative cost over 5 years incurred by 3 programs at different coverage levels for first-time mothers (N=193).^a^

Group, cost, coverage					Upfront (SGD)		First year (SGD)		Second year (SGD)		Third year (SGD)		Fourth year (SGD)		Fifth year (SGD)
**Web-based intervention group**															
	**Program cost**														
			10%, 50%, 100%	17,210		20,110		23,011		25,911		28,811		31,711	
	**Health care cost**														
			10%	0		679,800		1,359,600		2,039,400		2,719,200		3,399,000	
			50%	0		3,399,000		6,798,000		10,197,000		13,596,000		16,995,000	
			100%	0		6,798,000		13,596,000		20,394,000		27,192,000		33,990,000	
	**Total cost**														
			10%	17,210		699,910		1,382,611		2,065,311		2,748,011		3,430,711	
			50%	17,210		3,419,110		6,821,011		10,222,911		13,624,811		17,026,711	
			100%	17,210		6,818,110		13,619,011		20,419,911		27,220,811		34,021,711	
**Home-based intervention group**															
		**Program cost**													
			10%	233		155,073		309,913		464,753		619,593		774,433	
			50%	233		774,433		1,548,633		2,322,833		3,097,033		3,871,233	
			100%	233		1,548,633		3,097,033		4,645,433		6,193,833		7,742,233	
		**Health care cost**													
			10%	0		750,800		1,501,600		2,252,400		3,003,200		3,754,000	
			50%	0		3,754,000		7,508,000		11,262,000		15,016,000		18,770,000	
			100%	0		7,508,000		15,016,000		22,524,000		30,032,000		37,540,000	
		**Total cost**													
			10%	233		905,873		1,811,513		2,717,153		3,622,793		4,528,433	
			50%	233		4,528,433		9,056,633		13,584,833		18,113,033		22,641,233	
			100%	233		9,056,633		18,113,033		27,169,433		36,225,833		45,282,233	
**Control group**															
		**Program cost**													
			10%, 50%, 100%	0		0		0		0		0		0	
		**Health care cost**													
			10%	0		822,400		1,644,800		2,467,200		3,289,600		4,112,000	
			50%	0		4,112,000		8,224,000		12,336,000		16,448,000		20,560,000	
			100%	0		8,224,000		16,448,000		24,672,000		32,896,000		41,120,000	
		**Total cost**													
			10%	0		822,400		1,644,800		2,467,200		3,289,600		4,112,000	
			50%	0		4,112,000		8,224,000		12,336,000		16,448,000		20,560,000	
			100%	0		8,224,000		16,448,000		24,672,000		32,896,000		41,120,000	

^a^SGD 1=USD 0.75.

## Discussion

### Principal Results

To the best of our knowledge, this is the first study that economically evaluated modes of delivering psychoeducation to first-time mothers. Data on economic outcome measures were less frequently reported [34]. Findings from this study indicated that the web-based psychoeducation program was the most cost-effective approach compared to the home-based and control programs. In the base-case analysis, the web-based psychoeducation program dominated the other 2 programs across all outcomes. It saved around SGD 81 per participant (18% of the average adjusted cost) compared to the home-based program and around SGD 41 per participant compared to the routine care alone while showing better performance over them. Our findings are consistent with the results from previous studies demonstrating that web-based or internet-based intervention is cost-effective for various populations when compared to routine care or home-based intervention [10,11,19,20]. In addition, our findings are in line with results from systematic reviews and meta-analyses of the effectiveness and cost-effectiveness of eHealth interventions in somatic diseases, in which most studies indicated that eHealth was effective and cost-effective or at least promising [34]. Another systematic review and meta-analysis reporting the effectiveness and cost-effectiveness of eHealth interventions for depression and anxiety in primary care demonstrated that eHealth was only cost-effective for depression, but there was no evidence for its effectiveness for anxiety [35]. The findings are partly inconsistent with ours, which demonstrated that the web-based program was more cost-effective than the home-based and control programs for self-efficacy, social support, depression, and anxiety. In our sensitivity analysis, we also noticed that the probability of being the best cost-effective was between 45% and 50% for all 4 reported outcomes (Figure 5). Hence, our findings provided evidence that the web-based approach may be a cost-effective way of delivering psychoeducational interventions but need further confirmation with large scale data (eg, real-world study) [36].

To evaluate the budget impact of adopting the programs, we used the average cost data and the estimated number of first-time mothers in Singapore to project the cumulative cost over 5 years [11]. Assuming a 10% to 100% coverage of the web-based psychoeducation program, we found that around SGD 1 million to SGD 11.2 million would be saved compared to the home-based program or around SGD 680,000 to SGD 7.1 million costs would be saved when compared to the control program at the fifth year. The tremendous projected savings for the web-based program make it promising and attractive to be implemented nationwide. In short, over the 5-year projection, the web-based program showed long-term cost-savings compared to the home-based program and the routine care. Although the upfront cost was high for the web-based program, the low maintenance fee made it superior to the other 2 groups.

### Strengths and Limitations

This study has several strengths, which include its rigorous study design and the use of market data for personnel costs. The cost-effectiveness analysis was conducted alongside the 3-group repeated measures randomized controlled trial with a 6-month follow-up period. To evaluate the robustness of the results, we conducted both deterministic and probabilistic sensitivity analyses to check the uncertainty of our model. When the overall program cost was increased by 2-3 times (200% and 300% of the overall program costs, respectively), the control group cost the least among the 3 programs. However, the ICER of the web-based program over control program was rather low, that is, ranging from SGD 1.50 to SGD 6.50 per score improvement among all outcomes for 200% of the overall program cost and from SGD 9.60 to SGD 41.60 per score improvement among all outcomes for 300% of the overall program cost. The web-based program could be a preferred approach, given these relatively low ICERs. The cost-effectiveness acceptability curves were constructed to assess the sampling uncertainty from the trial based on 10,000 times bootstrapped results. The probabilities of the web-based program being cost-effective increased when the willingness-to-pay threshold increased and remained the highest among all 3 groups regardless of the willingness-to-pay thresholds. Despite the strengths, there were some limitations in this study. First, medical utilization data were collected at an individual level for all participants during the follow-up period and captured all possible costs incurred. However, the sample size was estimated based on the clinical effectiveness of the primary outcome of self-efficacy rather than the cost-effectiveness; thus, this might have contributed to uncertainty surrounding the results [37]. Meanwhile, there were 11 participants with missing cost data for the analysis, which might have affected the estimation of the cost for each group. To better address the uncertainty, we conducted a series of deterministic and probabilistic sensitivity analyses and found that the results were quite consistent and robust across the various analyses. In addition, this study used a randomized controlled trial with a 3-group study design, and the effectiveness of the interventions was evaluated up to 6 months after childbirth (about 5 months after the intervention), thus contributing to the robust study design [12,16] and ensuring that the findings from this study were representative and meaningful. Second, this analysis estimated costs from a health care perspective and not the estimated costs from a societal perspective. Therefore, it is recommended that future studies should conduct cost-effectiveness analysis of similar intervention from a social perspective. Third, the measurements of the health services utilization were based on self-reports at each follow-up data collection, which might lead to an underestimation of health resource use in comparison with daily recording in diaries, as people may forget to recall the services used [10]. However, in this study, the recall periods were kept short. The recall period for the costs of health resource used was only 3 months. Moreover, since we used a randomized design in this study, this underestimation was likely to be equally distributed among the 3 groups. Therefore, it is unlikely that the ICERS were affected in this study. Lastly, the calculated costs of the projection of nationwide implementation of interventions over 5 years were based on all first-time mothers, including those with complications. Since we excluded these mothers with severe complications in this study, our estimated costs might be conservative since the actual health care costs for those with complications might be higher. However, this would not affect the group comparison outcomes.

### Clinical Implications and Recommendations for Future Studies

Costs, clinical considerations, and participant preferences are often used to determine the choice of a treatment or intervention and its delivery [38]. The findings from this study suggest that a web-based postnatal psychoeducational intervention for first-time mothers was the most cost-effective approach for all outcomes measured. Currently, such postnatal psychoeducational interventions are not routinely provided in Singapore and need to be seriously considered in postnatal care to support mothers, especially first-time mothers. In addition, future studies using diaries to record the cost of using health resources and estimating costs from both health care and societal perspectives are recommended.

### Conclusions

Our findings suggest that compared to home-based psychoeducational intervention and routine care, the web-based approach was promisingly cost-effective to deliver the postnatal psychoeducational intervention to first-time mothers and could be adopted by hospitals as part of postnatal care support.

## Abbreviations

ICER: incremental cost-effectiveness ratio

## References

[1] Cowan CP, Cowan PA (2000). When Partners Become Parents: The Big Life Change for Couples.

[2] Murray L, Arteche A, Fearon P, Halligan S, Goodyer I, Cooper P (2011). Maternal postnatal depression and the development of depression in offspring up to 16 years of age. J Am Acad Child Adolesc Psychiatry.

[3] Rubin R (1967). Attainment of the maternal role. Part I: Processes. Nurs Res.

[4] Forster DA, McLachlan HL, Rayner J, Yelland J, Gold L, Rayner S (2008). The early postnatal period: exploring women's views, expectations and experiences of care using focus groups in Victoria, Australia. BMC Pregnancy Childbirth.

[5] Ong SF, Chan WS, Shorey S, Chong YS, Klainin-Yobas P, He H (2014). Postnatal experiences and support needs of first-time mothers in Singapore: a descriptive qualitative study. Midwifery.

[6] Shorey S, Chan SWC, Chong YS, He H (2015). A randomized controlled trial of the effectiveness of a postnatal psychoeducation programme on self-efficacy, social support and postnatal depression among primiparas. J Adv Nurs.

[7] Rowe HJ, Fisher JR (2010). Development of a universal psycho-educational intervention to prevent common postpartum mental disorders in primiparous women: a multiple method approach. BMC Public Health.

[8] Shorey S, Chan WS, Chong YS, He H (2015). A randomized controlled trial of the effectiveness of a postnatal psychoeducation programme on outcomes of primiparas: study protocol. J Adv Nurs.

[9] Drost RMWA, Paulus ATG, Jander AF, Mercken L, de Vries Hein, Ruwaard D, Evers SMAA (2016). A web-based computer-tailored alcohol prevention program for adolescents: Cost-effectiveness and intersectoral costs and benefits. J Med Internet Res.

[10] Larsen B, Marcus B, Pekmezi D, Hartman S, Gilmer T (2017). A web-based physical activity intervention for Spanish-speaking Latinas: A costs and cost-effectiveness analysis. J Med Internet Res.

[11] Internet users in the world. Internet World Stats.

[12] He H, Zhu L, Chan SWC, Chong Y, Jiao N, Chan YH, Luo N, Shorey S (2018). The Effectiveness and Cost-Effectiveness of Web-Based and Home-Based Postnatal Psychoeducational Interventions for First-Time Mothers: Randomized Controlled Trial Protocol. JMIR Res Protoc.

[13] (2017). Home access programme to benefit 8,000 low income households. Infocomm Media Development Authority of Singapore.

[14] McNamee P, Murray E, Kelly MP, Bojke L, Chilcott J, Fischer A, West R, Yardley L (2016). Designing and Undertaking a Health Economics Study of Digital Health Interventions. Am J Prev Med.

[15] Schechter CB, Walker EA, Ortega FM, Chamany S, Silver LD (2016). Costs and effects of a telephonic diabetes self-management support intervention using health educators. J Diabetes Complications.

[16] Jiao N, Zhu L, Chong YS, Chan WS, Luo N, Wang W, Hu R, Chan YH, He H (2019). Web-based versus home-based postnatal psychoeducational interventions for first-time mothers: A randomised controlled trial. Int J Nurs Stud.

[17] Wantland DJ, Portillo CJ, Holzemer WL, Slaughter R, McGhee EM (2004). The effectiveness of Web-based vs. non-Web-based interventions: a meta-analysis of behavioral change outcomes. J Med Internet Res.

[18] Shorey S, Lau Y, Dennis C, Chan YS, Tam WWS, Chan YH (2017). A randomized-controlled trial to examine the effectiveness of the 'Home-but not Alone' mobile-health application educational programme on parental outcomes. J Adv Nurs.

[19] Rollo ME, Burrows T, Vincze LJ, Harvey J, Collins CE, Hutchesson MJ (2018). Cost evaluation of providing evidence-based dietetic services for weight management in adults: In-person versus eHealth delivery. Nutr Diet.

[20] Li J, Parrott S, Sweeting M, Farmer A, Ross J, Dack C, Pal K, Yardley L, Barnard M, Hudda M, Alkhaldi G, Murray E (2018). Cost-Effectiveness of Facilitated Access to a Self-Management Website, Compared to Usual Care, for Patients With Type 2 Diabetes (HeLP-Diabetes): Randomized Controlled Trial. J Med Internet Res.

[21] Wong BB, Chan YH, Leow MQH, Lu Y, Chong YS, Koh SSL, He H (2017). Application of cabbage leaves compared to gel packs for mothers with breast engorgement: Randomised controlled trial. Int J Nurs Stud.

[22] Bandura A (1997). Self-efficacy: The Exercise of Control.

[23] Azmoude E, Jafarnejade F, Mazlom S (2015). The predictors for maternal self-efficacy in early parenthood. J Midwifery Reprod Health.

[24] Kohlhoff J, Barnett B (2013). Parenting self-efficacy: links with maternal depression, infant behaviour and adult attachment. Early Hum Dev.

[25] Leahy-Warren P, McCarthy G, Corcoran P (2012). First-time mothers: social support, maternal parental self-efficacy and postnatal depression. J Clin Nurs.

[26] Ngai F, Chan SWC, Ip W (2010). Predictors and correlates of maternal role competence and satisfaction. Nurs Res.

[27] Barnes CR, Adamson-Macedo EN (2007). Perceived Maternal Parenting Self-Efficacy (PMP S-E) tool: development and validation with mothers of hospitalized preterm neonates. J Adv Nurs.

[28] Shorey S, Chan SW, Chong YS, He H (2014). Maternal parental self-efficacy in newborn care and social support needs in Singapore: a correlational study. J Clin Nurs.

[29] Johansson K, Darj E (2004). What type of information do parents need after being discharged directly from the delivery ward?. Ups J Med Sci.

[30] Lee AM, Lam SK, Sze Mun Lau Stephanie Marie, Chong CSY, Chui HW, Fong DYT (2007). Prevalence, course, and risk factors for antenatal anxiety and depression. Obstet Gynecol.

[31] Bjelland I, Dahl AA, Haug TT, Neckelmann D (2002). The validity of the Hospital Anxiety and Depression Scale. An updated literature review. J Psychosom Res.

[32] Drummond MF, Sculpher MJ, Claxton K, Stoddart GL, Torrance GW (2015). Methods for the Economic Evaluation of Health Care Programmes (4th edn).

[33] (2017). Singapore report on registration of births and deaths. Immigration and Checkpoints Authority of Singapore.

[34] Elbert NJ, van Os-Medendorp Harmieke, van Renselaar Wilco, Ekeland AG, Hakkaart-van Roijen Leona, Raat H, Nijsten TEC, Pasmans SGMA (2014). Effectiveness and cost-effectiveness of ehealth interventions in somatic diseases: a systematic review of systematic reviews and meta-analyses. J Med Internet Res.

[35] Massoudi B, Holvast F, Bockting CLH, Burger H, Blanker MH (2019). The effectiveness and cost-effectiveness of e-health interventions for depression and anxiety in primary care: A systematic review and meta-analysis. J Affect Disord.

[36] Mihalopoulos C, Vos T, Pirkis J, Carter R (2012). The population cost-effectiveness of interventions designed to prevent childhood depression. Pediatrics.

[37] Willan AR, O'Brien BJ (1999). Sample size and power issues in estimating incremental cost-effectiveness ratios from clinical trials data. Health Econ.

[38] Hall SM, Lightwood JM, Humfleet GL, Bostrom A, Reus VI, Muñoz Ricardo (2005). Cost-effectiveness of bupropion, nortriptyline, and psychological intervention in smoking cessation. J Behav Health Serv Res.

